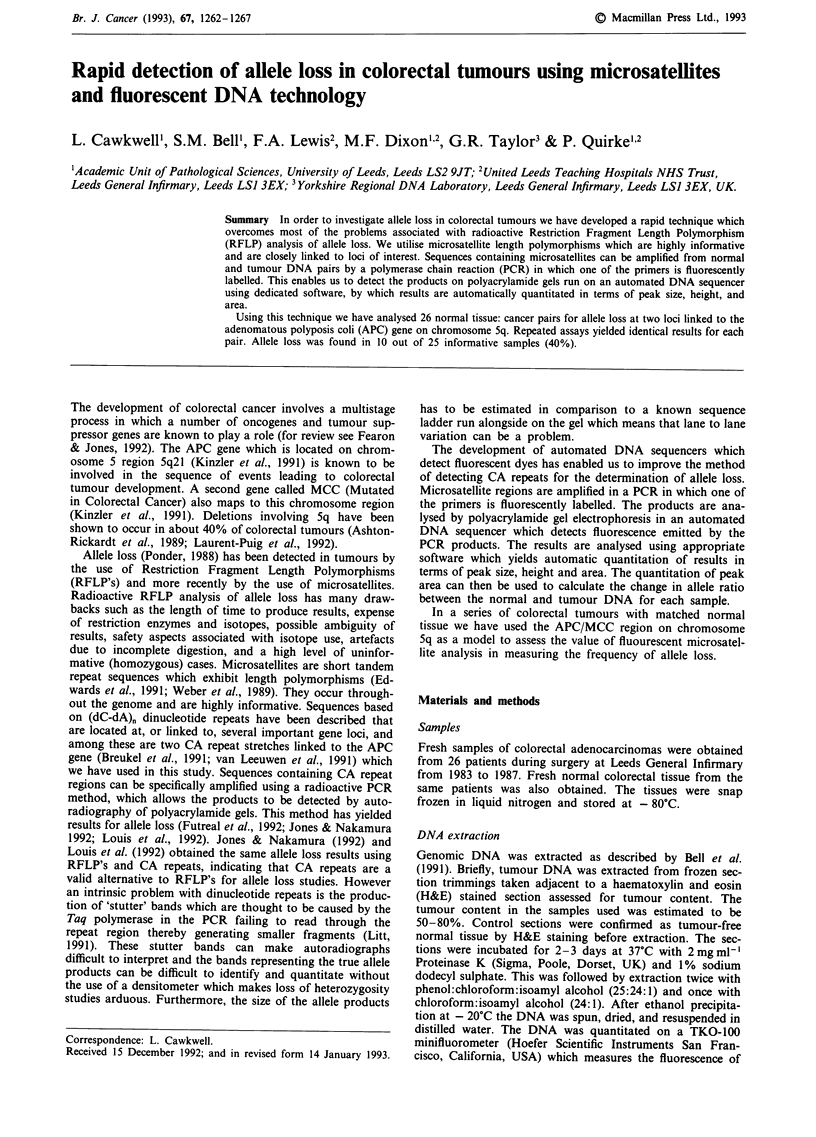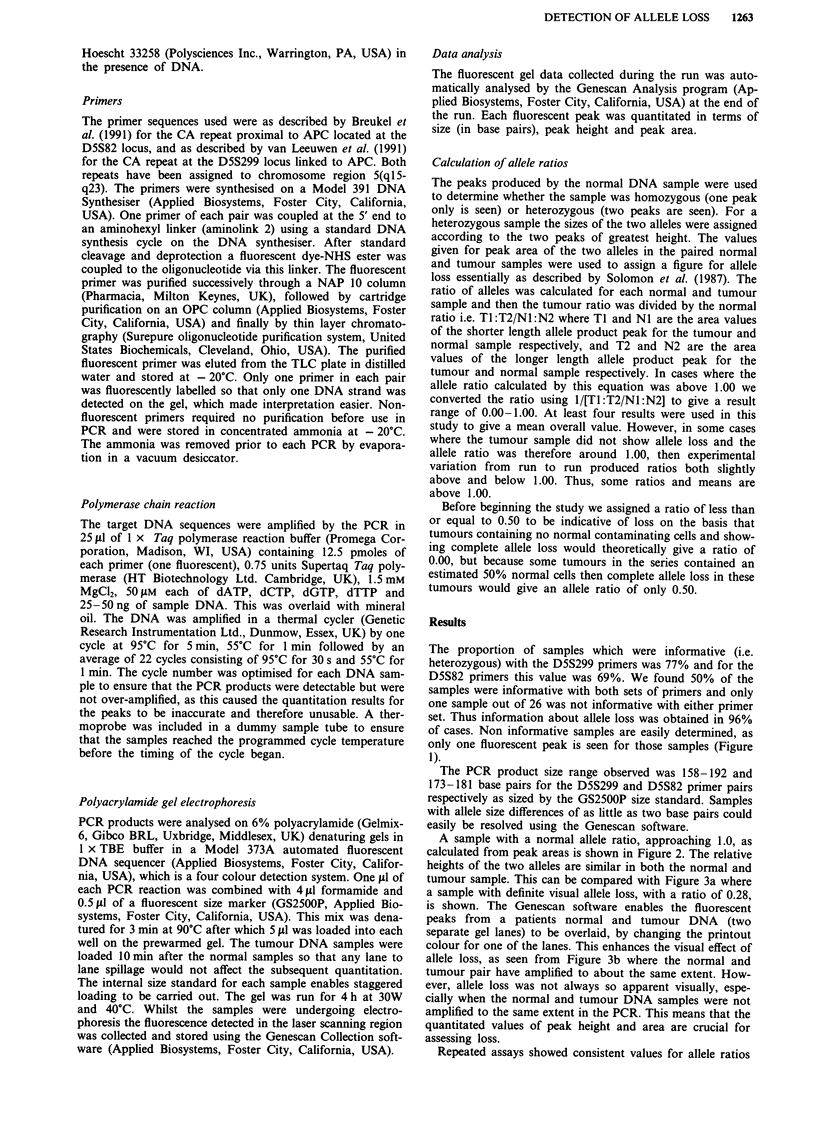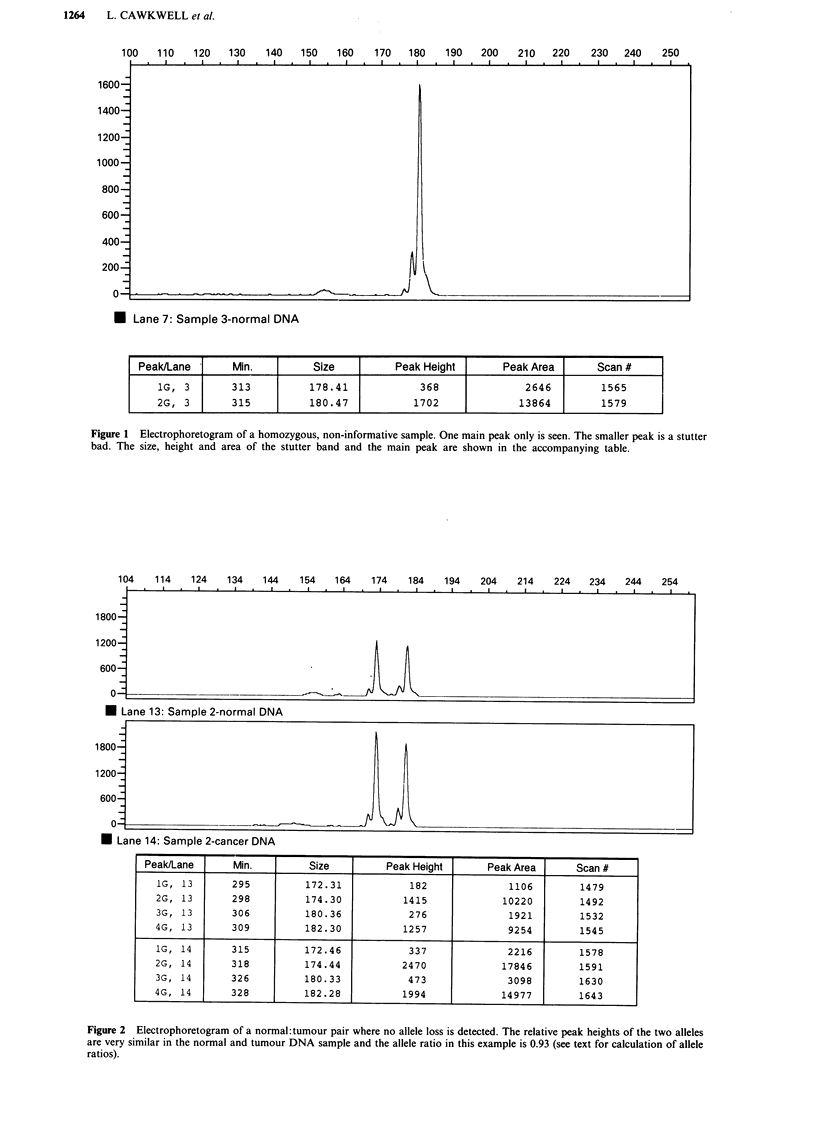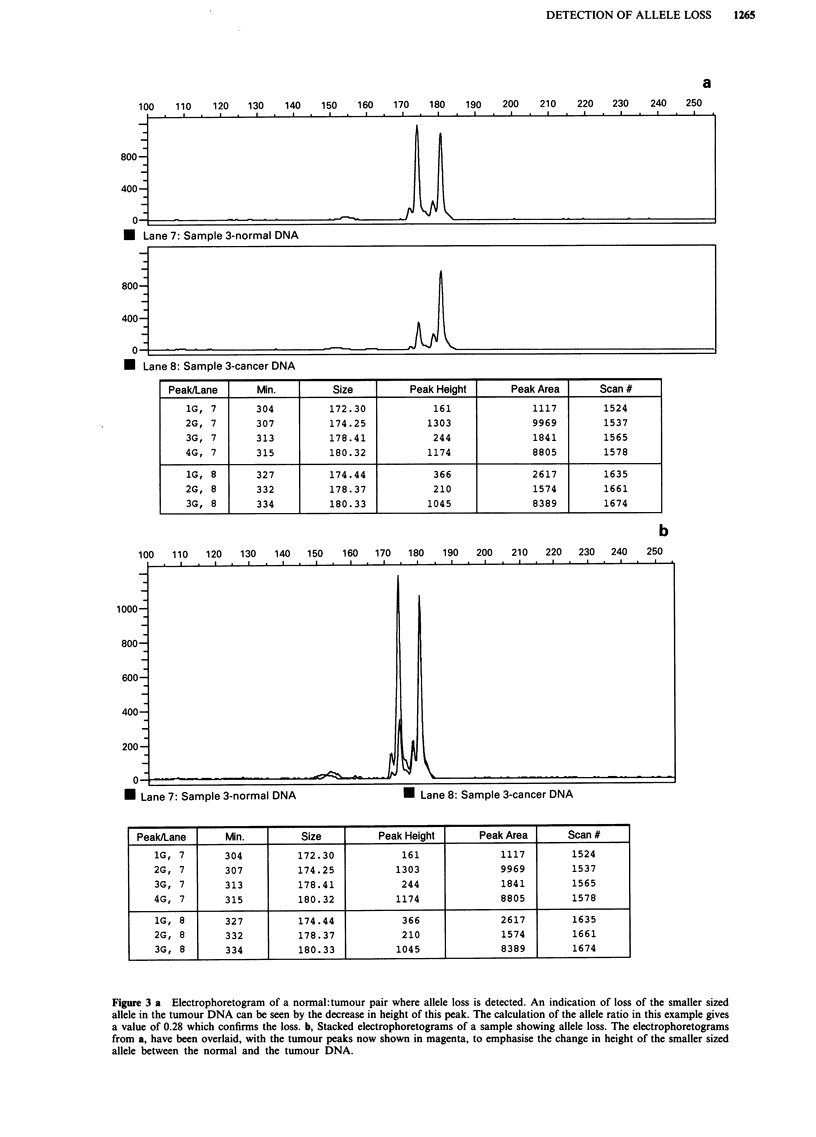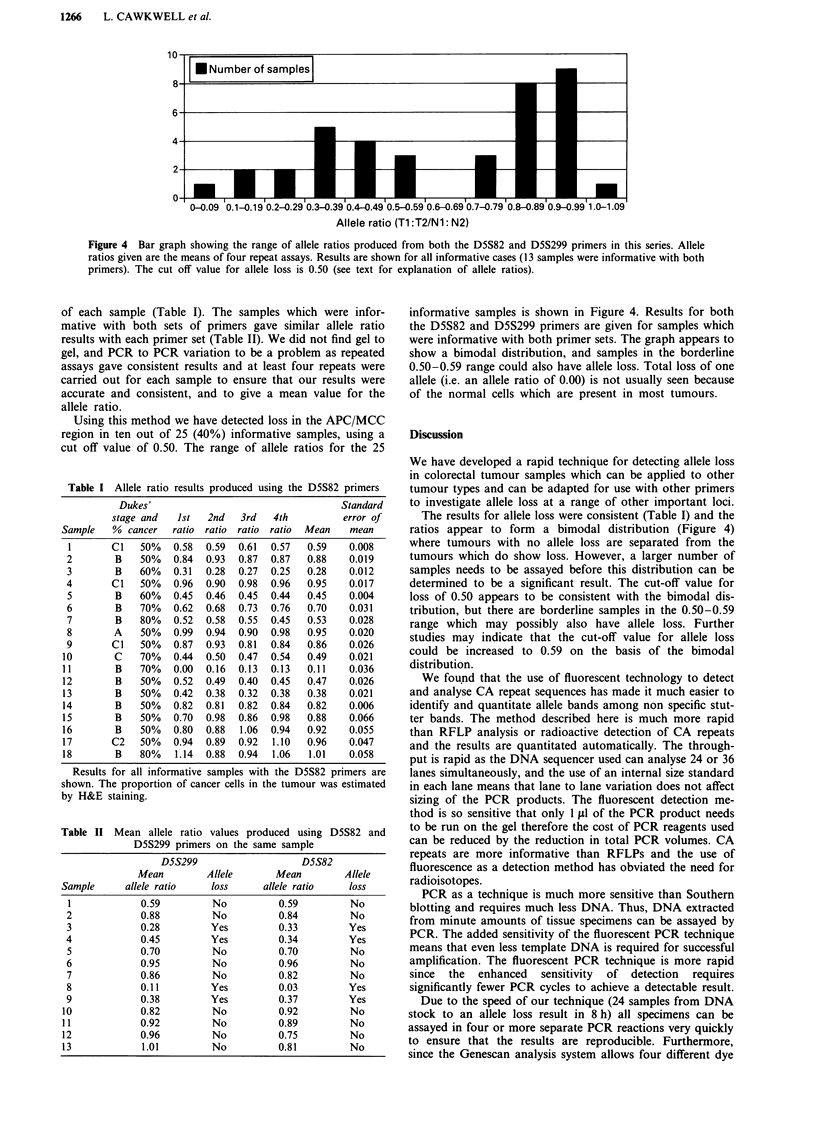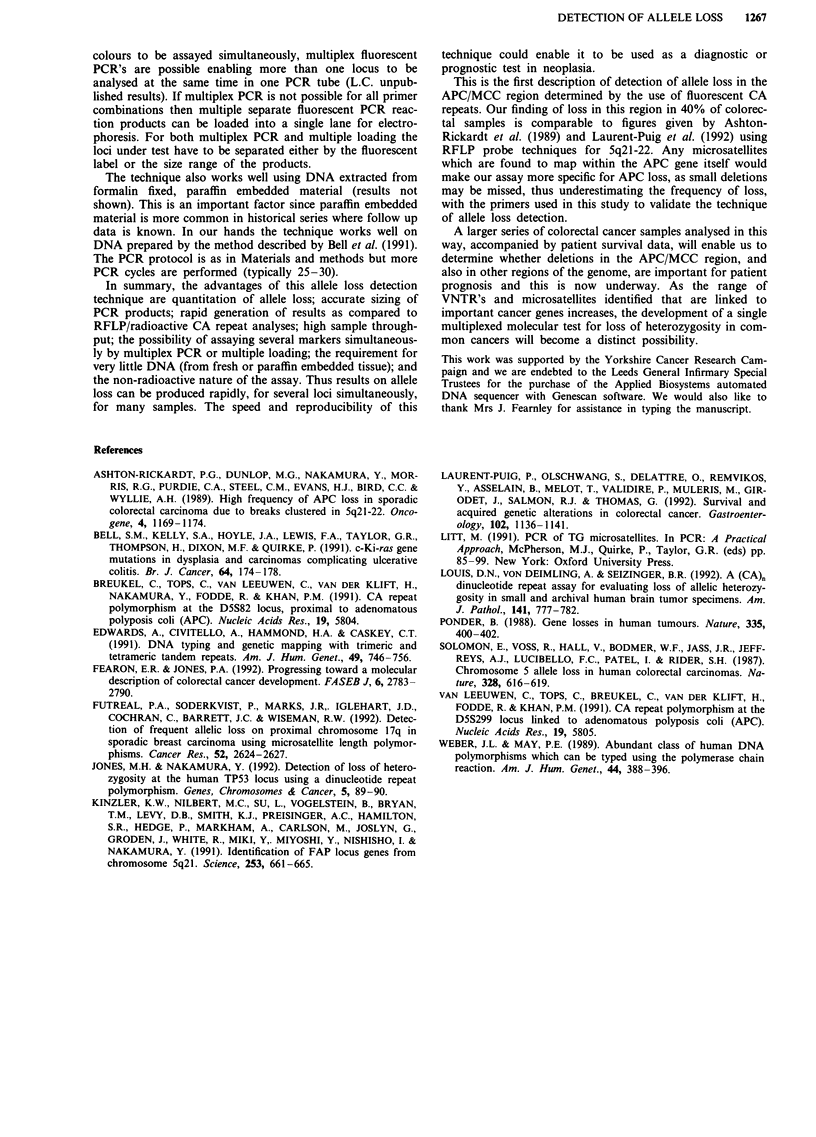# Rapid detection of allele loss in colorectal tumours using microsatellites and fluorescent DNA technology.

**DOI:** 10.1038/bjc.1993.236

**Published:** 1993-06

**Authors:** L. Cawkwell, S. M. Bell, F. A. Lewis, M. F. Dixon, G. R. Taylor, P. Quirke

**Affiliations:** Academic Unit of Pathological Sciences, University of Leeds, UK.

## Abstract

In order to investigate allele loss in colorectal tumours we have developed a rapid technique which overcomes most of the problems associated with radioactive Restriction Fragment Length Polymorphism (RFLP) analysis of allele loss. We utilise microsatellite length polymorphisms which are highly informative and are closely linked to loci of interest. Sequences containing microsatellites can be amplified from normal and tumour DNA pairs by a polymerase chain reaction (PCR) in which one of the primers is fluorescently labelled. This enables us to detect the products on polyacrylamide gels run on an automated DNA sequencer using dedicated software, by which results are automatically quantitated in terms of peak size, height, and area. Using this technique we have analysed 26 normal tissue: cancer pairs for allele loss at two loci linked to the adenomatous polyposis coli (APC) gene on chromosome 5q. Repeated assays yielded identical results for each pair. Allele loss was found in 10 out of 25 informative samples (40%).


Br. J. Cancer (1993), 67, 1262-1267 © Macmillan Press Ltd., 1993

### Rapid detection of allele loss in colorectal tumours using microsatellites and fluorescent DNA technology

L. Cawkwell[1], S.M. Bell[1], F.A. Lewis[2], M.F. Dixon[1,2], G.R. Taylor[3] & P. Quirke[1,2]

[1]*Academic Unit of Pathological Sciences, University of Leeds, Leeds LS2 9JT;* [2]*United Leeds Teaching Hospitals NHS Trust, Leeds General Infirmary, Leeds LS1 3EX;* [3]*Yorkshire Regional DNA Laboratory, Leeds General Infirmary, Leeds LS1 3EX, UK.*

**Summary** In order to investigate allele loss in colorectal tumours we have developed a rapid technique which overcomes most of the problems associated with radioactive Restriction Fragment Length Polymorphism (RFLP) analysis of allele loss. We utilise microsatellite length polymorphisms which are highly informative and are closely linked to loci of interest. Sequences containing microsatellites can be amplified from normal and tumour DNA pairs by a polymerase chain reaction (PCR) in which one of the primers is fluorescently labelled. This enables us to detect the products on polyacrylamide gels run on an automated DNA sequencer using dedicated software, by which results are automatically quantitated in terms of peak size, height, and area.

Using this technique we have analysed 26 normal tissue: cancer pairs for allele loss at two loci linked to the adenomatous polyposis coli (APC) gene on chromosome 5q. Repeated assays yielded identical results for each pair. Allele loss was found in 10 out of 25 informative samples (40%).

The development of colorectal cancer involves a multistage process in which a number of oncogenes and tumour suppressor genes are known to play a role (for review see Fearon & Jones, 1992). The APC gene which is located on chromosome 5 region 5q21 (Kinzler *et al.*, 1991) is known to be involved in the sequence of events leading to colorectal tumour development. A second gene called MCC (Mutated in Colorectal Cancer) also maps to this chromosome region (Kinzler *et al.*, 1991). Deletions involving 5q have been shown to occur in about 40% of colorectal tumours (Ashton-Rickardt *et al.*, 1989; Laurent-Puig *et al.*, 1992).

Allele loss (Ponder, 1988) has been detected in tumours by the use of Restriction Fragment Length Polymorphisms (RFLP's) and more recently by the use of microsatellites. Radioactive RFLP analysis of allele loss has many drawbacks such as the length of time to produce results, expense of restriction enzymes and isotopes, possible ambiguity of results, safety aspects associated with isotope use, artefacts due to incomplete digestion, and a high level of uninformative (homozygous) cases. Microsatellites are short tandem repeat sequences which exhibit length polymorphisms (Edwards *et al.*, 1991; Weber *et al.*, 1989). They occur throughout the genome and are highly informative. Sequences based on $(dC\text{-}dA)_n$ dinucleotide repeats have been described that are located at, or linked to, several important gene loci, and among these are two CA repeat stretches linked to the APC gene (Breukel *et al.*, 1991; van Leeuwen *et al.*, 1991) which we have used in this study. Sequences containing CA repeat regions can be specifically amplified using a radioactive PCR method, which allows the products to be detected by autoradiography of polyacrylamide gels. This method has yielded results for allele loss (Futreal *et al.*, 1992; Jones & Nakamura 1992; Louis *et al.*, 1992). Jones & Nakamura (1992) and Louis *et al.* (1992) obtained the same allele loss results using RFLP's and CA repeats, indicating that CA repeats are a valid alternative to RFLP's for allele loss studies. However an intrinsic problem with dinucleotide repeats is the production of 'stutter' bands which are thought to be caused by the *Taq* polymerase in the PCR failing to read through the repeat region thereby generating smaller fragments (Litt, 1991). These stutter bands can make autoradiographs difficult to interpret and the bands representing the true allele products can be difficult to identify and quantitate without the use of a densitometer which makes loss of heterozygosity studies arduous. Furthermore, the size of the allele products has to be estimated in comparison to a known sequence ladder run alongside on the gel which means that lane to lane variation can be a problem.

The development of automated DNA sequencers which detect fluorescent dyes has enabled us to improve the method of detecting CA repeats for the determination of allele loss. Microsatellite regions are amplified in a PCR in which one of the primers is fluorescently labelled. The products are analysed by polyacrylamide gel electrophoresis in an automated DNA sequencer which detects fluorescence emitted by the PCR products. The results are analysed using appropriate software which yields automatic quantitation of results in terms of peak size, height and area. The quantitation of peak area can then be used to calculate the change in allele ratio between the normal and tumour DNA for each sample.

In a series of colorectal tumours with matched normal tissue we have used the APC/MCC region on chromosome 5q as a model to assess the value of fluourescent microsatellite analysis in measuring the frequency of allele loss.

Correspondence: L. Cawkwell.
Received 15 December 1992; and in revised form 14 January 1993.

### Materials and methods

### *Samples*

Fresh samples of colorectal adenocarcinomas were obtained from 26 patients during surgery at Leeds General Infirmary from 1983 to 1987. Fresh normal colorectal tissue from the same patients was also obtained. The tissues were snap frozen in liquid nitrogen and stored at $-80°C$.

### *DNA extraction*

Genomic DNA was extracted as described by Bell *et al.* (1991). Briefly, tumour DNA was extracted from frozen section trimmings taken adjacent to a haematoxylin and eosin (H&E) stained section assessed for tumour content. The tumour content in the samples used was estimated to be 50-80%. Control sections were confirmed as tumour-free normal tissue by H&E staining before extraction. The sections were incubated for 2-3 days at 37°C with $2\ mg\ ml^{-1}$ Proteinase K (Sigma, Poole, Dorset, UK) and 1% sodium dodecyl sulphate. This was followed by extraction twice with phenol:chloroform:isoamyl alcohol (25:24:1) and once with chloroform:isoamyl alcohol (24:1). After ethanol precipitation at $-20°C$ the DNA was spun, dried, and resuspended in distilled water. The DNA was quantitated on a TKO-100 minifluorometer (Hoefer Scientific Instruments San Francisco, California, USA) which measures the fluorescence of

Hoescht 33258 (Polysciences Inc., Warrington, PA, USA) in the presence of DNA.

*Primers*

The primer sequences used were as described by Breukel *et al.* (1991) for the CA repeat proximal to APC located at the D5S82 locus, and as described by van Leeuwen *et al.* (1991) for the CA repeat at the D5S299 locus linked to APC. Both repeats have been assigned to chromosome region 5(q15-q23). The primers were synthesised on a Model 391 DNA Synthesiser (Applied Biosystems, Foster City, California, USA). One primer of each pair was coupled at the 5′ end to an aminohexyl linker (aminolink 2) using a standard DNA synthesis cycle on the DNA synthesiser. After standard cleavage and deprotection a fluorescent dye-NHS ester was coupled to the oligonucleotide via this linker. The fluorescent primer was purified successively through a NAP 10 column (Pharmacia, Milton Keynes, UK), followed by cartridge purification on an OPC column (Applied Biosystems, Foster City, California, USA) and finally by thin layer chromatography (Surepure oligonucleotide purification system, United States Biochemicals, Cleveland, Ohio, USA). The purified fluorescent primer was eluted from the TLC plate in distilled water and stored at − 20°C. Only one primer in each pair was fluorescently labelled so that only one DNA strand was detected on the gel, which made interpretation easier. Non-fluorescent primers required no purification before use in PCR and were stored in concentrated ammonia at − 20°C. The ammonia was removed prior to each PCR by evaporation in a vacuum desiccator.

*Polymerase chain reaction*

The target DNA sequences were amplified by the PCR in 25 µl of 1 × *Taq* polymerase reaction buffer (Promega Corporation, Madison, WI, USA) containing 12.5 pmoles of each primer (one fluorescent), 0.75 units Supertaq *Taq* polymerase (HT Biotechnology Ltd. Cambridge, UK), 1.5 mM $MgCl_2$, 50 µM each of dATP, dCTP, dGTP, dTTP and 25–50 ng of sample DNA. This was overlaid with mineral oil. The DNA was amplified in a thermal cycler (Genetic Research Instrumentation Ltd., Dunmow, Essex, UK) by one cycle at 95°C for 5 min, 55°C for 1 min followed by an average of 22 cycles consisting of 95°C for 30 s and 55°C for 1 min. The cycle number was optimised for each DNA sample to ensure that the PCR products were detectable but were not over-amplified, as this caused the quantitation results for the peaks to be inaccurate and therefore unusable. A thermoprobe was included in a dummy sample tube to ensure that the samples reached the programmed cycle temperature before the timing of the cycle began.

*Polyacrylamide gel electrophoresis*

PCR products were analysed on 6% polyacrylamide (Gelmix-6, Gibco BRL, Uxbridge, Middlesex, UK) denaturing gels in 1 × TBE buffer in a Model 373A automated fluorescent DNA sequencer (Applied Biosystems, Foster City, California, USA), which is a four colour detection system. One µl of each PCR reaction was combined with 4 µl formamide and 0.5 µl of a fluorescent size marker (GS2500P, Applied Biosystems, Foster City, California, USA). This mix was denatured for 3 min at 90°C after which 5 µl was loaded into each well on the prewarmed gel. The tumour DNA samples were loaded 10 min after the normal samples so that any lane to lane spillage would not affect the subsequent quantitation. The internal size standard for each sample enables staggered loading to be carried out. The gel was run for 4 h at 30W and 40°C. Whilst the samples were undergoing electrophoresis the fluorescence detected in the laser scanning region was collected and stored using the Genescan Collection software (Applied Biosystems, Foster City, California, USA).

*Data analysis*

The fluorescent gel data collected during the run was automatically analysed by the Genescan Analysis program (Applied Biosystems, Foster City, California, USA) at the end of the run. Each fluorescent peak was quantitated in terms of size (in base pairs), peak height and peak area.

*Calculation of allele ratios*

The peaks produced by the normal DNA sample were used to determine whether the sample was homozygous (one peak only is seen) or heterozygous (two peaks are seen). For a heterozygous sample the sizes of the two alleles were assigned according to the two peaks of greatest height. The values given for peak area of the two alleles in the paired normal and tumour samples were used to assign a figure for allele loss essentially as described by Solomon *et al.* (1987). The ratio of alleles was calculated for each normal and tumour sample and then the tumour ratio was divided by the normal ratio i.e. T1:T2/N1:N2 where T1 and N1 are the area values of the shorter length allele product peak for the tumour and normal sample respectively, and T2 and N2 are the area values of the longer length allele product peak for the tumour and normal sample respectively. In cases where the allele ratio calculated by this equation was above 1.00 we converted the ratio using 1/[T1:T2/N1:N2] to give a result range of 0.00–1.00. At least four results were used in this study to give a mean overall value. However, in some cases where the tumour sample did not show allele loss and the allele ratio was therefore around 1.00, then experimental variation from run to run produced ratios both slightly above and below 1.00. Thus, some ratios and means are above 1.00.

Before beginning the study we assigned a ratio of less than or equal to 0.50 to be indicative of loss on the basis that tumours containing no normal contaminating cells and showing complete allele loss would theoretically give a ratio of 0.00, but because some tumours in the series contained an estimated 50% normal cells then complete allele loss in these tumours would give an allele ratio of only 0.50.

### Results

The proportion of samples which were informative (i.e. heterozygous) with the D5S299 primers was 77% and for the D5S82 primers this value was 69%. We found 50% of the samples were informative with both sets of primers and only one sample out of 26 was not informative with either primer set. Thus information about allele loss was obtained in 96% of cases. Non informative samples are easily determined, as only one fluorescent peak is seen for those samples (Figure 1).

The PCR product size range observed was 158–192 and 173–181 base pairs for the D5S299 and D5S82 primer pairs respectively as sized by the GS2500P size standard. Samples with allele size differences of as little as two base pairs could easily be resolved using the Genescan software.

A sample with a normal allele ratio, approaching 1.0, as calculated from peak areas is shown in Figure 2. The relative heights of the two alleles are similar in both the normal and tumour sample. This can be compared with Figure 3a where a sample with definite visual allele loss, with a ratio of 0.28, is shown. The Genescan software enables the fluorescent peaks from a patients normal and tumour DNA (two separate gel lanes) to be overlaid, by changing the printout colour for one of the lanes. This enhances the visual effect of allele loss, as seen from Figure 3b where the normal and tumour pair have amplified to about the same extent. However, allele loss was not always so apparent visually, especially when the normal and tumour DNA samples were not amplified to the same extent in the PCR. This means that the quantitated values of peak height and area are crucial for assessing loss.

Repeated assays showed consistent values for allele ratios

| Peak/Lane | Min. | Size | Peak Height | Peak Area | Scan # |
|---|---|---|---|---|---|
| 1G, 3 | 313 | 178.41 | 368 | 2646 | 1565 |
| 2G, 3 | 315 | 180.47 | 1702 | 13864 | 1579 |

**Figure 1** Electrophoretogram of a homozygous, non-informative sample. One main peak only is seen. The smaller peak is a stutter bad. The size, height and area of the stutter band and the main peak are shown in the accompanying table.

| Peak/Lane | Min. | Size | Peak Height | Peak Area | Scan # |
|---|---|---|---|---|---|
| 1G, 13 | 295 | 172.31 | 182 | 1106 | 1479 |
| 2G, 13 | 298 | 174.30 | 1415 | 10220 | 1492 |
| 3G, 13 | 306 | 180.36 | 276 | 1921 | 1532 |
| 4G, 13 | 309 | 182.30 | 1257 | 9254 | 1545 |
| 1G, 14 | 315 | 172.46 | 337 | 2216 | 1578 |
| 2G, 14 | 318 | 174.44 | 2470 | 17846 | 1591 |
| 3G, 14 | 326 | 180.33 | 473 | 3098 | 1630 |
| 4G, 14 | 328 | 182.28 | 1994 | 14977 | 1643 |

**Figure 2** Electrophoretogram of a normal:tumour pair where no allele loss is detected. The relative peak heights of the two alleles are very similar in the normal and tumour DNA sample and the allele ratio in this example is 0.93 (see text for calculation of allele ratios).

a

| Peak/Lane | Min. | Size | Peak Height | Peak Area | Scan # |
|---|---|---|---|---|---|
| 1G, 7 | 304 | 172.30 | 161 | 1117 | 1524 |
| 2G, 7 | 307 | 174.25 | 1303 | 9969 | 1537 |
| 3G, 7 | 313 | 178.41 | 244 | 1841 | 1565 |
| 4G, 7 | 315 | 180.32 | 1174 | 8805 | 1578 |
| 1G, 8 | 327 | 174.44 | 366 | 2617 | 1635 |
| 2G, 8 | 332 | 178.37 | 210 | 1574 | 1661 |
| 3G, 8 | 334 | 180.33 | 1045 | 8389 | 1674 |

b

| Peak/Lane | Min. | Size | Peak Height | Peak Area | Scan # |
|---|---|---|---|---|---|
| 1G, 7 | 304 | 172.30 | 161 | 1117 | 1524 |
| 2G, 7 | 307 | 174.25 | 1303 | 9969 | 1537 |
| 3G, 7 | 313 | 178.41 | 244 | 1841 | 1565 |
| 4G, 7 | 315 | 180.32 | 1174 | 8805 | 1578 |
| 1G, 8 | 327 | 174.44 | 366 | 2617 | 1635 |
| 2G, 8 | 332 | 178.37 | 210 | 1574 | 1661 |
| 3G, 8 | 334 | 180.33 | 1045 | 8389 | 1674 |

**Figure 3 a** Electrophoretogram of a normal:tumour pair where allele loss is detected. An indication of loss of the smaller sized allele in the tumour DNA can be seen by the decrease in height of this peak. The calculation of the allele ratio in this example gives a value of 0.28 which confirms the loss. **b**, Stacked electrophoretograms of a sample showing allele loss. The electrophoretograms from **a**, have been overlaid, with the tumour peaks now shown in magenta, to emphasise the change in height of the smaller sized allele between the normal and the tumour DNA.

**Figure 4** Bar graph showing the range of allele ratios produced from both the D5S82 and D5S299 primers in this series. Allele ratios given are the means of four repeat assays. Results are shown for all informative cases (13 samples were informative with both primers). The cut off value for allele loss is 0.50 (see text for explanation of allele ratios).

of each sample (Table I). The samples which were informative with both sets of primers gave similar allele ratio results with each primer set (Table II). We did not find gel to gel, and PCR to PCR variation to be a problem as repeated assays gave consistent results and at least four repeats were carried out for each sample to ensure that our results were accurate and consistent, and to give a mean value for the allele ratio.

Using this method we have detected loss in the APC/MCC region in ten out of 25 (40%) informative samples, using a cut off value of 0.50. The range of allele ratios for the 25 informative samples is shown in Figure 4. Results for both the D5S82 and D5S299 primers are given for samples which were informative with both primer sets. The graph appears to show a bimodal distribution, and samples in the borderline 0.50–0.59 range could also have allele loss. Total loss of one allele (i.e. an allele ratio of 0.00) is not usually seen because of the normal cells which are present in most tumours.

**Table I** Allele ratio results produced using the D5S82 primers

| *Sample* | *Dukes' stage and % cancer* | | *1st ratio* | *2nd ratio* | *3rd ratio* | *4th ratio* | *Mean* | *Standard error of mean* |
|---|---|---|---|---|---|---|---|---|
| 1 | C1 | 50% | 0.58 | 0.59 | 0.61 | 0.57 | 0.59 | 0.008 |
| 2 | B | 50% | 0.84 | 0.93 | 0.87 | 0.87 | 0.88 | 0.019 |
| 3 | B | 60% | 0.31 | 0.28 | 0.27 | 0.25 | 0.28 | 0.012 |
| 4 | C1 | 50% | 0.96 | 0.90 | 0.98 | 0.96 | 0.95 | 0.017 |
| 5 | B | 60% | 0.45 | 0.46 | 0.45 | 0.44 | 0.45 | 0.004 |
| 6 | B | 70% | 0.62 | 0.68 | 0.73 | 0.76 | 0.70 | 0.031 |
| 7 | B | 80% | 0.52 | 0.58 | 0.55 | 0.45 | 0.53 | 0.028 |
| 8 | A | 50% | 0.99 | 0.94 | 0.90 | 0.98 | 0.95 | 0.020 |
| 9 | C1 | 50% | 0.87 | 0.93 | 0.81 | 0.84 | 0.86 | 0.026 |
| 10 | C | 70% | 0.44 | 0.50 | 0.47 | 0.54 | 0.49 | 0.021 |
| 11 | B | 70% | 0.00 | 0.16 | 0.13 | 0.13 | 0.11 | 0.036 |
| 12 | B | 50% | 0.52 | 0.49 | 0.40 | 0.45 | 0.47 | 0.026 |
| 13 | B | 50% | 0.42 | 0.38 | 0.32 | 0.38 | 0.38 | 0.021 |
| 14 | B | 50% | 0.82 | 0.81 | 0.82 | 0.84 | 0.82 | 0.006 |
| 15 | B | 50% | 0.70 | 0.98 | 0.86 | 0.98 | 0.88 | 0.066 |
| 16 | B | 50% | 0.80 | 0.88 | 1.06 | 0.94 | 0.92 | 0.055 |
| 17 | C2 | 50% | 0.94 | 0.89 | 0.92 | 1.10 | 0.96 | 0.047 |
| 18 | B | 80% | 1.14 | 0.88 | 0.94 | 1.06 | 1.01 | 0.058 |

Results for all informative samples with the D5S82 primers are shown. The proportion of cancer cells in the tumour was estimated by H&E staining.

**Table II** Mean allele ratio values produced using D5S82 and D5S299 primers on the same sample

| | *D5S299* | | *D5S82* | |
|---|---|---|---|---|
| *Sample* | *Mean allele ratio* | *Allele loss* | *Mean allele ratio* | *Allele loss* |
| 1 | 0.59 | No | 0.59 | No |
| 2 | 0.88 | No | 0.84 | No |
| 3 | 0.28 | Yes | 0.33 | Yes |
| 4 | 0.45 | Yes | 0.34 | Yes |
| 5 | 0.70 | No | 0.70 | No |
| 6 | 0.95 | No | 0.96 | No |
| 7 | 0.86 | No | 0.82 | No |
| 8 | 0.11 | Yes | 0.03 | Yes |
| 9 | 0.38 | Yes | 0.37 | Yes |
| 10 | 0.82 | No | 0.92 | No |
| 11 | 0.92 | No | 0.89 | No |
| 12 | 0.96 | No | 0.75 | No |
| 13 | 1.01 | No | 0.81 | No |

### Discussion

We have developed a rapid technique for detecting allele loss in colorectal tumour samples which can be applied to other tumour types and can be adapted for use with other primers to investigate allele loss at a range of other important loci.

The results for allele loss were consistent (Table I) and the ratios appear to form a bimodal distribution (Figure 4) where tumours with no allele loss are separated from the tumours which do show loss. However, a larger number of samples needs to be assayed before this distribution can be determined to be a significant result. The cut-off value for loss of 0.50 appears to be consistent with the bimodal distribution, but there are borderline samples in the 0.50–0.59 range which may possibly also have allele loss. Further studies may indicate that the cut-off value for allele loss could be increased to 0.59 on the basis of the bimodal distribution.

We found that the use of fluorescent technology to detect and analyse CA repeat sequences has made it much easier to identify and quantitate allele bands among non specific stutter bands. The method described here is much more rapid than RFLP analysis or radioactive detection of CA repeats and the results are quantitated automatically. The throughput is rapid as the DNA sequencer used can analyse 24 or 36 lanes simultaneously, and the use of an internal size standard in each lane means that lane to lane variation does not affect sizing of the PCR products. The fluorescent detection method is so sensitive that only 1 μl of the PCR product needs to be run on the gel therefore the cost of PCR reagents used can be reduced by the reduction in total PCR volumes. CA repeats are more informative than RFLPs and the use of fluorescence as a detection method has obviated the need for radioisotopes.

PCR as a technique is much more sensitive than Southern blotting and requires much less DNA. Thus, DNA extracted from minute amounts of tissue specimens can be assayed by PCR. The added sensitivity of the fluorescent PCR technique means that even less template DNA is required for successful amplification. The fluorescent PCR technique is more rapid since the enhanced sensitivity of detection requires significantly fewer PCR cycles to achieve a detectable result.

Due to the speed of our technique (24 samples from DNA stock to an allele loss result in 8 h) all specimens can be assayed in four or more separate PCR reactions very quickly to ensure that the results are reproducible. Furthermore, since the Genescan analysis system allows four different dye colours to be assayed simultaneously, multiplex fluorescent PCR's are possible enabling more than one locus to be analysed at the same time in one PCR tube (L.C. unpublished results). If multiplex PCR is not possible for all primer combinations then multiple separate fluorescent PCR reaction products can be loaded into a single lane for electrophoresis. For both multiplex PCR and multiple loading the loci under test have to be separated either by the fluorescent label or the size range of the products.

The technique also works well using DNA extracted from formalin fixed, paraffin embedded material (results not shown). This is an important factor since paraffin embedded material is more common in historical series where follow up data is known. In our hands the technique works well on DNA prepared by the method described by Bell *et al.* (1991). The PCR protocol is as in Materials and methods but more PCR cycles are performed (typically 25–30).

In summary, the advantages of this allele loss detection technique are quantitation of allele loss; accurate sizing of PCR products; rapid generation of results as compared to RFLP/radioactive CA repeat analyses; high sample throughput; the possibility of assaying several markers simultaneously by multiplex PCR or multiple loading; the requirement for very little DNA (from fresh or paraffin embedded tissue); and the non-radioactive nature of the assay. Thus results on allele loss can be produced rapidly, for several loci simultaneously, for many samples. The speed and reproducibility of this technique could enable it to be used as a diagnostic or prognostic test in neoplasia.

This is the first description of detection of allele loss in the APC/MCC region determined by the use of fluorescent CA repeats. Our finding of loss in this region in 40% of colorectal samples is comparable to figures given by Ashton-Rickardt *et al.* (1989) and Laurent-Puig *et al.* (1992) using RFLP probe techniques for 5q21-22. Any microsatellites which are found to map within the APC gene itself would make our assay more specific for APC loss, as small deletions may be missed, thus underestimating the frequency of loss, with the primers used in this study to validate the technique of allele loss detection.

A larger series of colorectal cancer samples analysed in this way, accompanied by patient survival data, will enable us to determine whether deletions in the APC/MCC region, and also in other regions of the genome, are important for patient prognosis and this is now underway. As the range of VNTR's and microsatellites identified that are linked to important cancer genes increases, the development of a single multiplexed molecular test for loss of heterozygosity in common cancers will become a distinct possibility.

This work was supported by the Yorkshire Cancer Research Campaign and we are endebted to the Leeds General Infirmary Special Trustees for the purchase of the Applied Biosystems automated DNA sequencer with Genescan software. We would also like to thank Mrs J. Fearnley for assistance in typing the manuscript.

### References

ASHTON-RICKARDT, P.G., DUNLOP, M.G., NAKAMURA, Y., MORRIS, R.G., PURDIE, C.A., STEEL, C.M., EVANS, H.J., BIRD, C.C. & WYLLIE, A.H. (1989). High frequency of APC loss in sporadic colorectal carcinoma due to breaks clustered in 5q21-22. *Oncogene*, **4**, 1169–1174.

BELL, S.M., KELLY, S.A., HOYLE, J.A., LEWIS, F.A., TAYLOR, G.R., THOMPSON, H., DIXON, M.F. & QUIRKE, P. (1991). c-Ki-*ras* gene mutations in dysplasia and carcinomas complicating ulcerative colitis. *Br. J. Cancer*, **64**, 174–178.

BREUKEL, C., TOPS, C., VAN LEEUWEN, C., VAN DER KLIFT, H., NAKAMURA, Y., FODDE, R. & KHAN, P.M. (1991). CA repeat polymorphism at the D5S82 locus, proximal to adenomatous polyposis coli (APC). *Nucleic Acids Res.*, **19**, 5804.

EDWARDS, A., CIVITELLO, A., HAMMOND, H.A. & CASKEY, C.T. (1991). DNA typing and genetic mapping with trimeric and tetrameric tandem repeats. *Am. J. Hum. Genet.*, **49**, 746–756.

FEARON, E.R. & JONES, P.A. (1992). Progressing toward a molecular description of colorectal cancer development. *FASEB J*, **6**, 2783–2790.

FUTREAL, P.A., SODERKVIST, P., MARKS, J.R,. IGLEHART, J.D., COCHRAN, C., BARRETT, J.C. & WISEMAN, R.W. (1992). Detection of frequent allelic loss on proximal chromosome 17q in sporadic breast carcinoma using microsatellite length polymorphisms. *Cancer Res.*, **52**, 2624–2627.

JONES, M.H. & NAKAMURA, Y. (1992). Detection of loss of heterozygosity at the human TP53 locus using a dinucleotide repeat polymorphism. *Genes, Chromosomes & Cancer*, **5**, 89–90.

KINZLER, K.W., NILBERT, M.C., SU, L., VOGELSTEIN, B., BRYAN, T.M., LEVY, D.B., SMITH, K.J., PREISINGER, A.C., HAMILTON, S.R., HEDGE, P., MARKHAM, A., CARLSON, M., JOSLYN, G., GRODEN, J., WHITE, R., MIKI, Y,. MIYOSHI, Y., NISHISHO, I. & NAKAMURA, Y. (1991). Identification of FAP locus genes from chromosome 5q21. *Science*, **253**, 661–665.

LAURENT-PUIG, P., OLSCHWANG, S., DELATTRE, O., REMVIKOS, Y., ASSELAIN, B., MELOT, T., VALIDIRE, P., MULERIS, M., GIRODET, J., SALMON, R.J. & THOMAS, G. (1992). Survival and acquired genetic alterations in colorectal cancer. *Gastroenterology*, **102**, 1136–1141.

LITT, M. (1991). PCR of TG microsatellites. In PCR: *A Practical Approach*, McPherson, M.J., Quirke, P., Taylor, G.R. (eds) pp. 85–99. New York: Oxford University Press.

LOUIS, D.N., VON DEIMLING, A. & SEIZINGER, B.R. (1992). A $(CA)_n$ dinucleotide repeat assay for evaluating loss of allelic heterozygosity in small and archival human brain tumor specimens. *Am. J. Pathol.*, **141**, 777–782.

PONDER, B. (1988). Gene losses in human tumours. *Nature*, **335**, 400–402.

SOLOMON, E., VOSS, R., HALL, V., BODMER, W.F., JASS, J.R., JEFFREYS, A.J., LUCIBELLO, F.C., PATEL, I. & RIDER, S.H. (1987). Chromosome 5 allele loss in human colorectal carcinomas. *Nature*, **328**, 616–619.

VAN LEEUWEN, C., TOPS, C., BREUKEL, C., VAN DER KLIFT, H., FODDE, R. & KHAN, P.M. (1991). CA repeat polymorphism at the D5S299 locus linked to adenomatous polyposis coli (APC). *Nucleic Acids Res.*, **19**, 5805.

WEBER, J.L. & MAY, P.E. (1989). Abundant class of human DNA polymorphisms which can be typed using the polymerase chain reaction. *Am. J. Hum. Genet.*, **44**, 388–396.